# Role of Virtual iMRI in Glioblastoma Surgery: Advantages, Limitations, and Correlation with iCT and Brain Shift

**DOI:** 10.3390/brainsci15010035

**Published:** 2024-12-31

**Authors:** Erica Grasso, Francesco Certo, Mario Ganau, Giulio Bonomo, Giuseppa Fiumanò, Giovanni Buscema, Andrea Maugeri, Antonella Agodi, Giuseppe M. V. Barbagallo

**Affiliations:** 1 Department of Medical and Surgical Sciences and Advanced Technologies “G.F. Ingrassia”, Neurological Surgery, Policlinico “G. Rodolico-San Marco” University Hospital, University of Catania, 95124 Catania, Italy; egrasso360@gmail.com (E.G.); cicciocerto@yahoo.it (F.C.); dott.giuliobonomo@gmail.com (G.B.); 2 Interdisciplinary Research Center on Brain Tumors Diagnosis and Treatment, University of Catania, 95124 Catania, Italy; 3 Nuffield Department of Clinical Neurosciences, University of Oxford, Oxford OX1 2JD, UK; mario.ganau@alumni.harvard.edu; 4 Department of Radiology and Radiotherapy, Policlinico “G. Rodolico-San Marco” University Hospital, University of Catania, 95124 Catania, Italy; gfiuman@sirm.org; 5 Department of Anesthesia and Intensive Care, University Hospital Policlinico “G. Rodolico-San Marco”, 95123 Catania, Italy; buscemagiovanni@gmail.com; 6 Department of Medical and Surgical Sciences and Advanced Technologies “G.F. Ingrassia”, University of Catania, 95124 Catania, Italy; andrea.maugeri@unict.it (A.M.); agodia@unict.it (A.A.)

**Keywords:** brain shift, elastic image fusion, glioblastoma, intraoperative CT, rigid image fusion, Virtual iMRI, brain tumour surgery

## Abstract

**Background**: Elastic image fusion (EIF) using an intraoperative CT (iCT) scan may enhance neuronavigation accuracy and compensate for brain shift. **Objective**: To evaluate the safety and reliability of the EIF algorithm (Virtual iMRI Cranial 4.5, Brainlab AG, Munich Germany, for the identification of residual tumour in glioblastoma surgery. Moreover, the impact of brain shift on software reliability is assessed. **Methods**: This ambispective study included 80 patients with a diagnosis of glioblastoma. Pre-operative MRI was elastically fused with an intraoperative CT scan (BodyTom; Samsung-Neurologica, Danvers, MA, USA) acquired at the end of the resection. Diagnostic specificity and the sensitivity of each tool was determined. The impact of brain shift on residual tumour was statistically analysed. An analysis of accuracy was performed through Target Registration Error (TRE) measurement after rigid image fusion (RIF) and EIF. A qualitative evaluation of each Virtual MRI image (VMRI) was performed. **Results**: VMRI identified residual tumour in 26/80 patients (32.5%), confirmed by post-operative MRI (true positive). Of these, 5 cases were left intentionally due to DES-positive responses, 8 cases underwent near maximal or subtotal resection, and 13 cases were not detected by iCT. However, in the other 27/80 cases (33.8%), VMRI reported residual tumour that was present neither on iCT nor on post-operative MRI (false positive). i-CT showed a sensitivity of 56% and specificity of 100%; VMRI demonstrated a sensitivity of 100% and specificity of 50%. Spearman correlation analysis showed a moderate correlation between pre-operative volume and VMRI tumour residual. Moreover, tumour involving insula or infiltrating more than one lobe displayed higher median values (*p* = 0.023) of virtual residual tumour. A statistically significant reduction towards lower TRE values after EIF was observed for test structures. **Conclusions**: Virtual iMRI was proven to be a feasible option to detect residual tumour. Its integration within a multimodal imaging protocol may provide neurosurgeons with intraoperatively updated imaging.

## 1. Introduction

Brain shift represents a major source of inaccuracy in neuronavigation during the microsurgical removal of intracranial lesions [1,2,3]. Due to the heterogeneous nature of cerebral lesions [4] and the non-uniform distribution of brain shift [5,6], current rigid fusion (RIF) algorithms hardly deal with its complexity [7]. Indeed, linear fusion algorithms exclusively allow translations, rotations, scaling, and skewness to align datasets [8]. Recently, a novel tool for elastic image fusion (EIF), named Elements Virtual iMRI Cranial (Brainlab AG, Munich, Germany), has been developed to compensate for brain shift. Based on intraoperative imaging, this method elastically deforms pre-operative MRI data on intraoperative CT(iCT) imaging and updates the treatment plan [9]. To date, only a limited number of clinical studies have been reported using such a novel approach using either intraoperative MRI (iMR) [10,11,12] or the AIRO CT scanner [8,9,13,14,15]. Pilot studies reported significantly improved co-registration accuracy [8,10] and elastically updated DTI fibre tracts regarding intraoperative neuromonitoring and post-operative clinical status [11]. Conversely, the effectiveness of the EIF algorithm in visualising the residual tumour by elastically fusing intraoperative BodyTom CT images has not been explored yet.

Our work aimed to study two major aspects regarding the application of the algorithm in glioblastoma surgery. The first goal was to assess software accuracy to generate a “virtual” MRI and detect residual tumour compared to intraoperative CT images’ sensitivity and specificity. Secondly, the impact of the major factors causing brain shift [2,7,16,17] was statistically analysed. Finally, image fusion accuracy was quantified by Target Registration Error (TRE) measurement and compared with previous data reported in the literature.

## 2. Materials and Methods

### 2.1. Patient Enrolment and Eligibility Criteria

In this cohort ambispective study, 80 patients with a diagnosis of glioblastoma (GBM, 2021 WHO classification) were included. Cases from January 2020 to August 2022 were analysed retrospectively, while the prospective cohort included patients from September 2022 to January 2024. Given the nature of this technological aid to surgical resection, the authors did not consider it ethically acceptable to perform a pure randomisation, and, rather, opted for a quasi-experimental cohort design, with retrospective analysis and prospective validation. Inclusion and exclusion criteria are reported in Table 1.

### 2.2. Data Acquisition and Volumetric Analysis

Data collection included the following information:

- Age
- Side and anatomical localisation of tumour within the brain
- Size of craniotomy
- Head position
- Ventricular opening
- Volumetric assessment:
  - Pre- and post-operative MRI tumour volume
  - Necrosis/tumour ratio (NTR)
  - Extent of resection (EOTR)
- Geometrical measurements for analysis of image accuracy:
  - Bifrontal ventricular width (referred to as “Evan’s index” in the text)
  - Midline shift
  - Euclidean distance between anatomical landmarks after rigid image fusion (RIF) and elastic image fusion (EIF) algorithms.

Craniotomy was measured in the axial plane as the maximum distance between the external cortical bone edges. Patient positioning was classified as follows based on the angular tilt of head: 1: 0°, 2: 30°, 3: 45°, 4: 90°, 5: prone. Tumour location was classified as follows: group 1: tumours involving frontal lobe, group 2: tumours involving parietal lobe, group 3: tumours involving temporal lobe, group 4: tumours involving occipital lobe, group 5: tumours involving insula, group 6: tumours involving central core, group 7: tumours involving more than one lobe.

Volumes (expressed in cm^3^) measured on pre-operative MRI included the following:

- “tumour”: enhancing area on T1-weighted gadolinium (Gd)-enhanced sequence
- “necrosis”: non-enhancing region within the tumour on T1-weighted Gd-enhanced MRI

Necrosis/ tumour ratio (NTR) was calculated as proposed by Henker et al. [18].

$$\mathrm{NTR}=\frac{\mathrm{NECROSIS}}{T_{1}\mathrm{Vol}}$$

EOTR was then calculated using the Sanai and Berger [19] method and classified, according to the evidence-based recommendations proposed by Karschnia et al. [20], as ‘biopsy’, ‘resection’, ‘subtotal resection’, ‘near total resection’, ‘complete resection’, and ‘supramaximal resection”.

Virtual iMRI (VMRI) tumour residual was defined as the area not included in the resection cavity and refers to the total residual volume detected by the VMRI algorithm, encompassing both true positives and false positives. Quantitative volumetric measurements of these specific regions of interest (ROIs) were performed using a semi-automated contouring tool (Elements SmartBrush, Brainlab). All measurements were performed by the same researcher to rule out inter-observer variability. Additionally, measurements were double-checked in ten randomly selected cases by a researcher not involved in the original analysis to validate the results and assess inter-observer variability.

### 2.3. Intraoperative Multimodal Imaging Protocol

In all cases, an intraoperative multimodal imaging protocol previously described in [21,22] was applied; this included intraoperative CT (i-CT) (BodyTom; Samsung-Neurologica, Danvers, MA, USA), 5-Aminolevulinic acid (5-ALA) fluorescence, neuronavigation, and Intraoperative Neurophysiological Monitoring (IONM). Neuronavigation was conducted using a commercially available software (Cranial Navigation, Brainlab AG, Munich, Germany). The pre-operative dataset uploaded included at least a 3D T1-weighted gadolinium-enhancing MRI sequence. The first pre-operative post-contrast iCT was obtained after patient positioning to rigidly fuse it with the pre-operative MRI dataset. As resection was deemed to be complete, a second post-contrast iCT was acquired to estimate the extent of resection. If further resection proceeded due to evidence of residual tumour on iCT, a third post-contrast iCT was performed. At the end of surgical resection, VMRI was elastically fused to update the estimate extent of resection.

Quantitative evaluation of residual tumour was evaluated on post-operative T1-weighted gadolinium-enhancing MRI sequences acquired within 48 h.

### 2.4. Image Fusion Accuracy and Quantitation of the Target Registration Error

**Figure 1:**
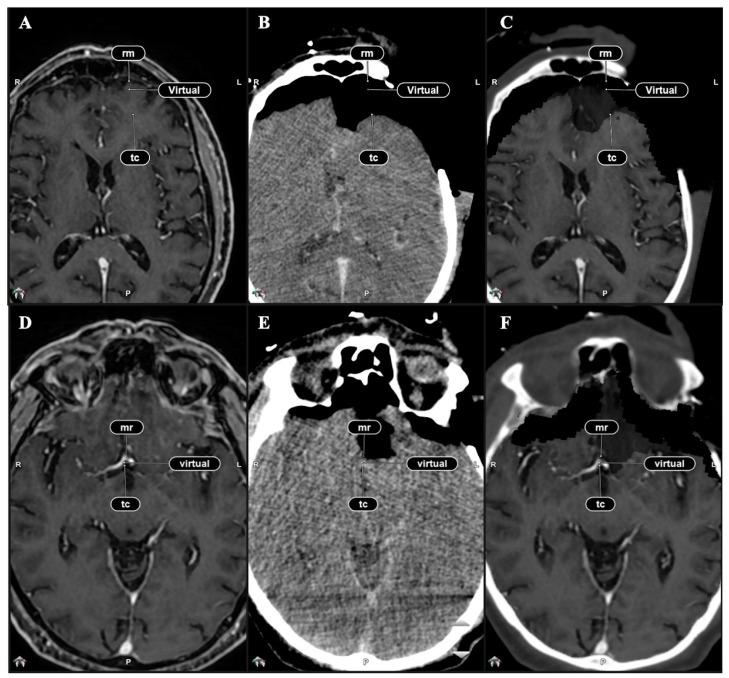
Identification of corresponding anatomic landmarks used for TRE calculation after RIF and EIF. (**A**) Pre-operative MRI with corresponding cortical points. (**B**) iCT scan image with corresponding cortical point. (**C**) Virtual iMRI with corresponding cortical points. (**D**) Pre-operative MRI with AComm set as reference point. (**E**) iCT scan image with corresponding AComm. (**F**) Virtual iMRI with corresponding AComm.

The spatial alignment of fused pre-operative and Virtual iMRI (VMRI) was assessed via Target Registration Error (TRE) measurements, which requires calculating Euclidean distance between manually defined landmarks [8,23,24,25,26,27]. In our study, the TREs between pre-operative MRI and iCT (after RIF) and between Virtual iMRI and iCT (after EIF), were measured as Euclidean distance between paired landmarks (Figure 1). Anterior–posterior and latero-lateral reference points were considered in our measurements and included the following anatomical structures:

- Maximum cortical displacement in lateral and cranio-caudal directions;
- Anterior communicating artery at its junction with A1 segment (AComm);
- Basilar apex;
- Midline shift;
- Evan’s index.

Anterior communicating artery (AComm) and Basilar apex were considered control structures, while cortical shift, midline shift, and Evan’s index were the test structures considered as parameters of cortical, parenchymal, and ventricular deformation, respectively. To better quantify the effect of brain shift on ventricular structures, in the statistical analysis, only the maximum bifrontal width of ventricles based on previous Evan’s index was considered.

### 2.5. Qualitative Comparison of iCT, Virtual iMRI, and Post-Operative MRI

**Figure 2:**
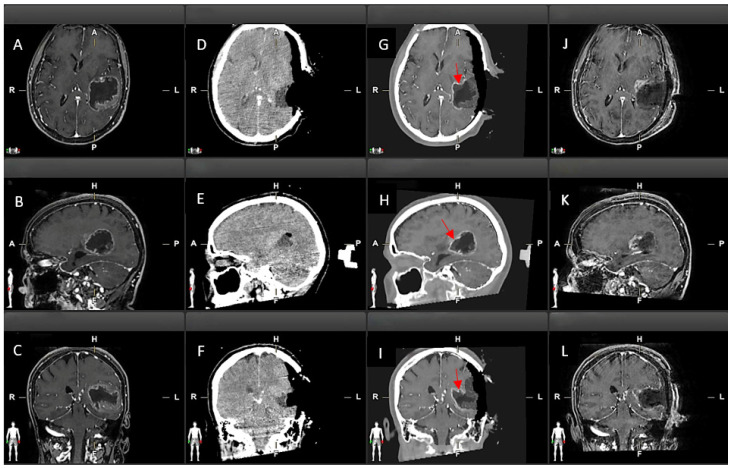
Method adopted for assessing the accuracy of Virtual iMRI. (**A**–**C**) Pre-operative MRI in axial, sagittal, and coronal view. (**D**–**F**) Post-contrast i-CT scan in axial, sagittal, and coronal view. (**G**–**I**) Virtual iMRI image with axial, sagittal, and coronal view. (**J**–**L**) Post-operative MRI in axial, sagittal, and coronal view. Red arrows indicate residual tumour on Virtual iMRI which is not included in the resection cavity (grey area).

Two expert neurosurgeons reviewed six different parameters resulting from EIF using a side-by-side approach (Figure 2). The observers primarily compared the VMRI images to the iCT data and, secondarily, VMRI and post-operative MRI. More specifically, factors analysed in the comparison between i-CT and Virtual iMRI were matching of relevant anatomical structures, residual tumour on i-CT, and matching of residual tumour on both images. Similarly, the analysis between VMRI and post-operative MR included matching of residual tumour, matching of surgical cavity edges, and overall quality of virtual image.

Accuracy was rated on a scale ranging from 1 to 5: 1, matching < 10 slices in at least 2 projections; 2, matching between 10 and 20 slices in at least 2 projections; 3, matching between 10 and 20 slices in the 3 projections; 4, matching > 20 slices in the 3 projections; 5, complete matching. Overall quality was rated as 1, low quality; 2, low-medium quality; 3, intermediate; 4, medium-high quality; 5, high quality.

### 2.6. Statistical Analysis

Data analysis was conducted using SPSS (version 29.0; IBM). The normality of the data and the suitability for parametric tests were assessed using the Shapiro–Wilk test. Descriptive statistics summarised quantitative variables using mean and standard error or median and interquartile range (IQR) based on their distribution (Table 2).

Spearman correlation analysis explored relationships among seven phenomena (residual tumour after EIF, lateral and cranio-caudal cortical shift, midline shift variation, Evan’s index variation, Basilar apex shift, AComm shift) and three parameters (craniotomy, tumour volume measured on pre-operative T1 gadolinium-enhancing MRI sequences, and NTR). Correlations were expressed as Spearman rho coefficients and associated *p*-values. Correlation strength was categorised according to Critical Values for Spearman’s Rank Order Correlation. Differences in the seven phenomena, categorised by ventricular opening, tumour localisation, and patient positioning, were analysed using either the Mann–Whitney or Kruskal–Wallis test.

Unadjusted linear regression models evaluated parameters potentially associated with residual tumour after EIF, with craniotomy, pre-operative T1, gadolinium enhancing, tumour volume, NTR, ventricular opening, tumour location, and patient positioning as independent variables. Adjusted linear regression analysis was applied for variables exhibiting significant associations in unadjusted models. β coefficients along with their standard error (SE) quantified the strength and direction of associations, and the coefficient of determination (R²) assessed the proportion of variance explained by independent variables. Comparison of lateral and cranio-caudal shift, anterior communicating artery, Basilar apex, Evan’s index, and midline shift after rigid and elastic fusion, as well as TRE quantities, was conducted using the Wilcoxon signed-rank test for paired samples. Due to the exploratory nature of the analyses, aiming to identify potential trends and associations rather than confirmatory testing, adjustments for multiple comparisons were not applied. A significance level of 0.05 and, if necessary, a 95% confidence interval, were applied to all analyses.

## 3. Results

### 3.1. Residual Tumour

Residual tumour was detected on post-operative MRI in 26 out of 80 patients (32.5%). Among these, intraoperative CT (iCT) correctly identified residual tumour in 17 patients (21.2%) and led to further resection in 12 cases, resulting in complete (4), near-complete (4), and subtotal (4) resections. In the other five cases, further resection could not be performed because the lesion was located in eloquent brain areas, or it showed positive responses to direct electrical stimulation (DES). In the remaining 13 out of 80 cases (16.3%), iCT failed to detect the residual tumour (false negative). No false positives were observed. Therefore, iCT showed 56% sensitivity and 100% specificity.

By contrast, VMRI identified residual tumour in 26/80 patients (32.5%), further confirmed by post-operative MRI (true positive). Of these, 5 cases were left intentionally due to DES-positive responses, 8 cases underwent near maximal or subtotal resection, and 13 cases were not detected in the iCT. However, in the other 27/80 cases (33.8%), VMRI reported residual tumour that was present neither on iCT nor on post-operative MRI (false positive). No false negatives were observed. Considering these results, Virtual iMRI demonstrated 100% sensitivity and 50% specificity (Table 3).

**Figure 3:**
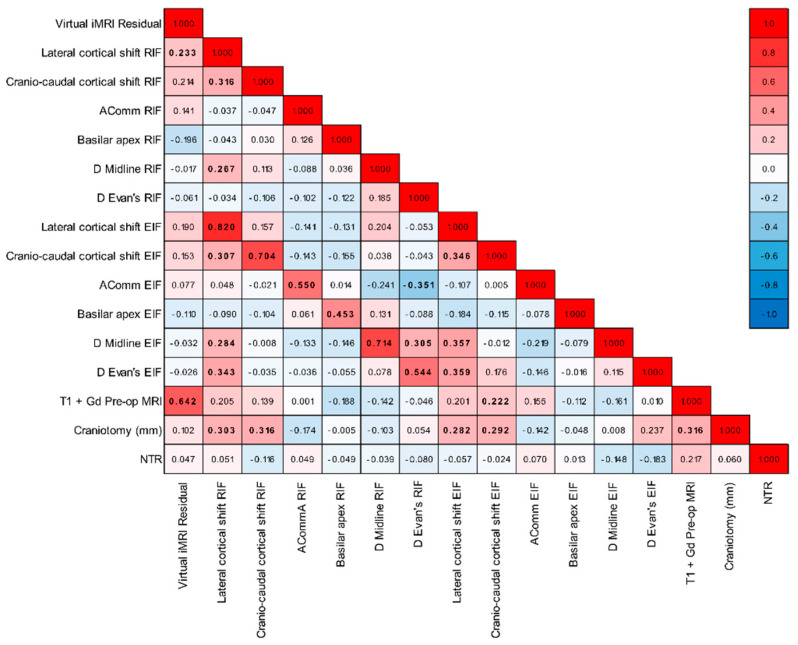
Heatmap illustrating the correlations among the quantitative variables under investigation. The figure presents Spearman correlation coefficients ranging from −1 (depicted in blue) to +1 (depicted in red). Correlations that are statistically significant (*p*-values < 0.05) are highlighted in bold.

**Figure 4:**
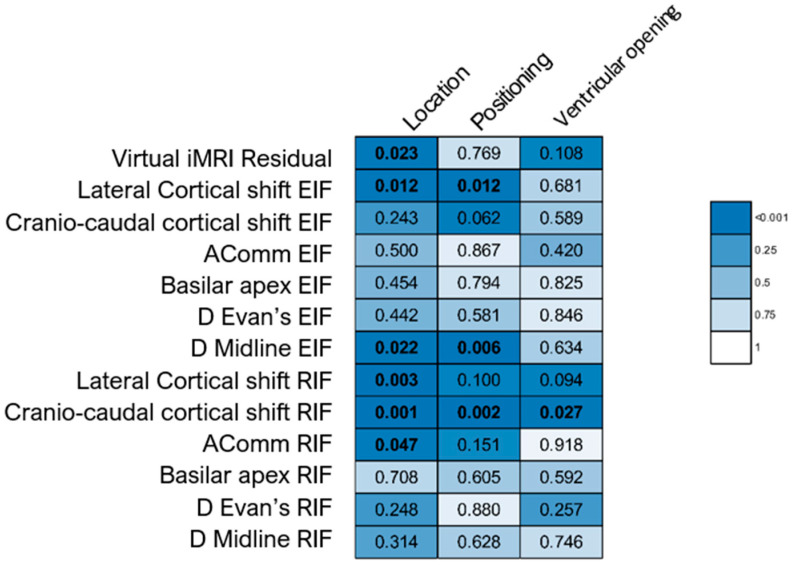
Heatmap illustrating the differences among the quantitative variables according to tumour location, patient positioning, and ventricular opening. The figure displays the *p*-values obtained through Mann–Whitney or Kruskal–Wallis tests. Statistically significant *p*-values (<0.05) are highlighted in bold.

Figure 3 and Figure 4 depict the results of bivariate analyses for all parameters under examination.

**Figure 5:**
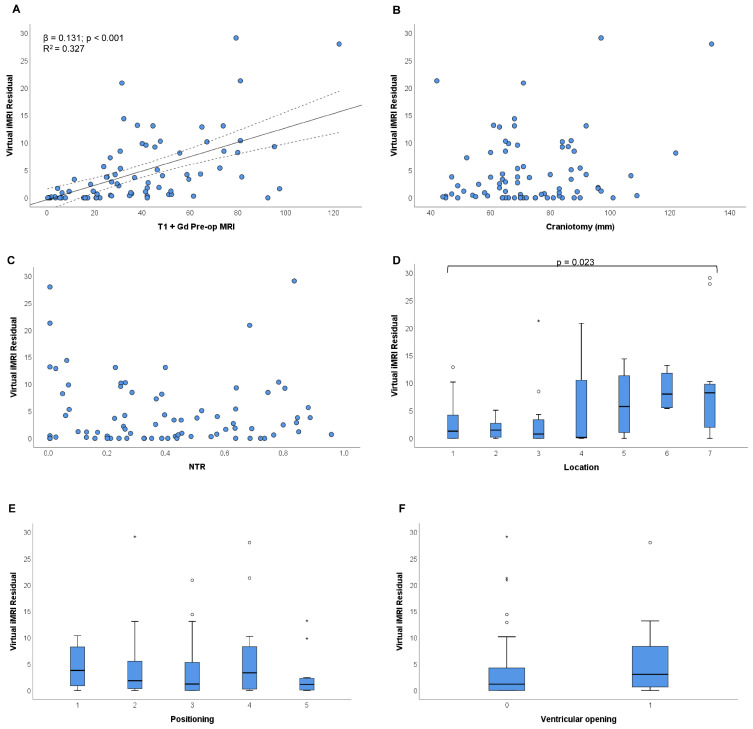
Factors potentially associated with residual tumour on Virtual iMRI. Scatter plots in Figures (**A**–**C**) show the relationship of Virtual iMRI residual with pre-operative T1 + Gd tumour volume, craniotomy, and NTR; in Figure (**A**), the solid line and dashed lines represent the linear regression line and its confidence intervals, respectively, for the statistically significant relationship between Virtual iMRI residual and pre-operative T1 + Gd tumour volume. Box plots in Figures (**D**–**F**) illustrate the variations in Virtual iMRI residual based on tumour location, patient positioning, and ventricular opening; the *p*-value in Figure (**D**) is determined by the Kruskal–Wallis test.

A moderate positive correlation was observed between VMRI tumour residual and pre-operative gadolinium-enhanced MRI (rho = 0.642; *p* < 0.001). The β coefficient from the simple linear regression suggests that, with each one-unit increase in pre-operative gadolinium-enhanced MRI, the VMRI tumour residual is expected to increase by 0.131 units. The R2 value of 0.327 suggests that approximately 32.7% of the variability in the dependent variable is explained by the independent variable in the model (Figure 5A). This means that the model accounts for a moderate proportion of the total variability in the dependent variable. Conversely, craniotomy and NTR showed non-significant correlations (rho = 0.102, *p* = 0.370 and rho= 0.047, *p* = 0.682, respectively). The absence of a linear relationship is corroborated by the scatter plots depicted in Figure 5B,C.

Variations in VMRI tumour residual were observable across various tumour locations (*p* = 0.023), particularly displaying higher median values within groups 5–7 (Figure 5D). No significant differences in VMRI residual were observed concerning head position (*p* = 0.769) and ventricular opening (*p* = 0.108) (Figure 5E,F).

The results from the adjusted linear regression model confirm the association between pre-operative gadolinium-enhanced MRI and tumour location with Virtual iMRI residual. More precisely, the VMRI residual increases by 0.120 units for each one-unit increment in pre-operative gadolinium-enhanced MRI (β = 0.120; SE = 0.020; *p* < 0.001). Furthermore, individuals belonging to tumour location groups 5–7 demonstrate higher values compared to those in other groups (β = 3.710; SE = 1.165; *p* = 0.002). The R2 increased compared to the unadjusted model, indicating that approximately 40.5% of the variability in the dependent variable is explained by the adjusted model.

### 3.2. Analysis on Accuracy: Quantitation of the Target Registration Error

For descriptive purposes, mean TRE values of paired anatomical landmarks measured between pre-operative MRI and iCT (after RIF) and between Virtual iMRI and iCT (after EIF) are summarised in Table 4.

**Figure 6:**
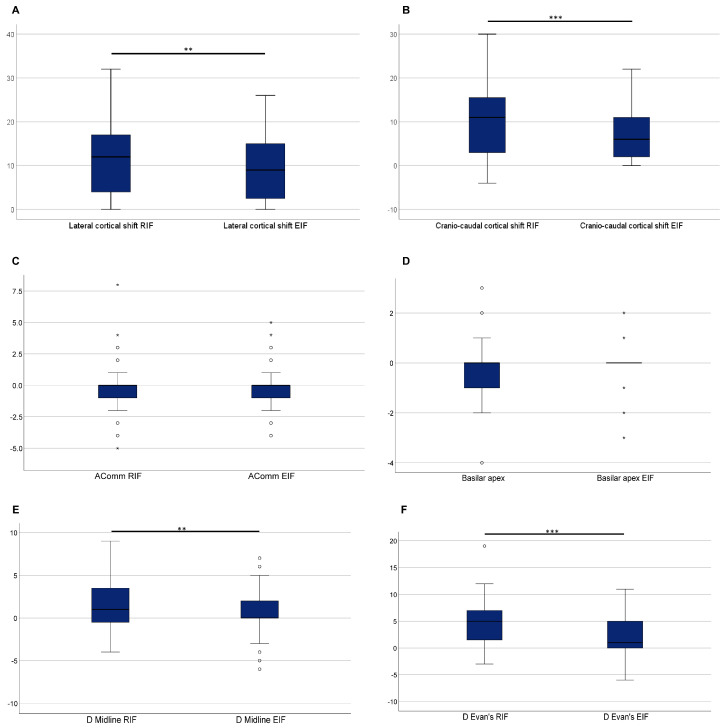
Comparison of lateral (**A**) and cranio-caudal shift (**B**), anterior communicating artery (**C**), Basilar apex (**D**), midline shift (**E**), and Evan’s index (**F**) after rigid and elastic fusion. Statistical significance is denoted as ** for *p*-value < 0.01, and *** for *p*-value < 0.001, based on the Wilcoxon signed-rank test for paired samples.

Figure 6 shows the comparison of lateral and cranio-caudal shift, anterior communicating artery, Basilar apex, Evan’s index, and midline shift after rigid and elastic fusion. Compared to post-RIF evaluation, significantly lower values after EIF were evident for lateral and cranio-caudal shift (*p* = 0.001 and *p* < 0.001, respectively), Evan’s index (*p* < 0.001), and midline shift (*p* = 0.008). Conversely, no significant reduction was observed for control structures (AComm and Basilar apex) after EIF (*p* = 0.169 and *p* = 0.218, respectively).

Concerning lateral and cranio-caudal cortical shift, certain correlations and differences were observed. While lateral cortical shift displayed weak correlations with craniotomy (rho = 0.282; *p* = 0.012), cranio-caudal cortical shift demonstrated weak correlations with both T1-enhanced MRI (rho = 0.222; *p* = 0.049) and craniotomy (rho = 0.292; *p* = 0.009). The lateral cortical shift also varied based on tumour location and positioning (*p*-values = 0.012), indicating significant pairwise differences between groups 1 and 5 of tumour location (*p* = 0.007) and groups 4 and 5 of positioning (*p* = 0.029). Comparable findings emerged when assessing the values after RIF, revealing moderate correlations between craniotomy and both lateral and cranio-caudal cortical shift, along with significant differences by tumour location, positioning, and ventricular opening (Figure 3 and Figure 4). Midline shift also exhibited differences based on tumour location and positioning (*p* = 0.022 and 0 = 0.006, respectively; Figure 3). Specifically, significant differences were observed between groups 1 and 5 for location (*p* = 0.049) and between pairs 1–4 and 2–4 for positioning (*p* = 0.007 and *p* = 0.046, respectively).

### 3.3. Qualitative Evaluation

On average, the matching of relevant anatomical structures between VMRI data and iCT scan resulted in score of 3.5 and approximately 3.3 regarding the matching of residual tumour. Similarly, the analysis between VMRI and post-operative MRI concerning the matching of residual tumour and the matching of surgical cavity edges showed a mean score of 3.2 and 3.3, respectively. Overall, quality image yielded a mean score of 3.4 meaning that, on average, VMRI accuracy was rated as intermediate. The evaluation performed in ten randomly selected cases was double-checked by a researcher not involved in the original analysis, which confirmed the absence of significant inter-observer variability in the qualitative evaluation of images.

## 4. Discussion

We described the use of a novel EIF algorithm to detect residual tumour and to update intraoperative neuronavigation. Unlike RIF algorithms, this tool deforms the preoperative plan, taking into account several physical forces such as gravity, cerebrospinal-fluid-related force, and the modelling of the collision of FEM-voxels, both with other soft- or stiff-tissue-related FEM-Voxels and with the rigid skull [8].

Compared to the few studies in the literature, ours provides new insights for different reasons. In previous studies, either iMR [10,11,12] or the AIRO CT scanner [8,9,13,14,15] were used. To the best of our knowledge, this is the first study reporting the results of intraoperative BodyTom CT images elastically fused using Virtual iMRI.

We have illustrated the diagnostic sensitivity and specificity of these two techniques (BodyTom i-CT and Virtual iMRI) in visualising residual tumour. As reported above, iCT showed 56% sensitivity and 100% specificity. Conversely, Virtual iMRI demonstrated 100% sensitivity and 50% specificity. These results suggest that the integration of this novel algorithm into the intraoperative workflow can offer the possibility of combining maximal iCT specificity with the higher sensitivity of EIF algorithm. Recently, Mazzucchi et al. [13] reported a sensitivity of 1 and a specificity of 0.33 of Virtual iMRI in glioblastoma surgery. However, the heterogeneity of the cohort, with only nine glioblastoma cases, and the absence of any analysis on the causes of false positives prevent us from comparing results. Conversely, we analysed the known factors causing brain shift to elucidate potential explanations for the large number of false positives. The results obtained from the adjusted linear regression model showed that EIF algorithm tends to report higher “virtual” residual tumour, which is quantitatively influenced mainly by the pre-operative volume and by tumour location, with tumour involving insula or infiltrating more than one lobe carrying the major brain shift effects. These results led us to hypothesise that smaller virtual residual tumours might be caused by errors occurring in the semi-automatic registration phase, whereas, in bigger tumours, the algorithm failed to include tumour into the resection cavity due to the major influence of brain shift. Compared to our study, Nimsky et al. [7] also documented the role of craniotomy size, resection volume, patient position, and tissue characteristics on brain shift after RIF using iMRI. As already stated by Hartkens et al. [28], we agree that the combination of these factors, rather than their isolated contribution, eventually determines the final result. Further studies with a larger cohort of patients will be needed to better assess how the interplay between these factors causes the EIF algorithm to crash.

Considering our results, surgeons should be cautious in planning the further resection of any eventual “virtual” residual tumour, especially when an increased risk of “false positive” virtual residual tumour can be suspected (i.e., high pre-operative tumour volume, insula or multiple lobe infiltration). In such cases, resorting to other intraoperative tools might settle the doubts between a “false positive” and a “true positive” residual. Several additional devices might help the surgeon. In 2014, Stummer et al. [29] demonstrated that 5-ALA fluorescence predicted solid and infiltrating tumour and concluded that fluorescence appears superior to contrast enhancement on MRI for indicating residual tumour. More recently, Roder et al. [30], in a nonrandomised prospective controlled trial (PCT), demonstrated the non-superiority of iMRI compared to 5-ALA, which might be advantageous economically and timing-wise given the significantly longer operating room times of iMRI. Intraoperative ultrasound (iUS) has been widely accepted as a real-time image-guided tool for the excision of intracranial lesions [3,30,31,32]. Moreover, in 2020, Della Pepa et al. [33] achieved the best results in terms of the extent of resection through a combination of both techniques, where the 5-ALA-guided procedure is followed by a final survey with iUS. In our institutional experience, the multimodal intraoperative imaging protocol based on the combination of intraoperative Ultrasound (iUS), intraoperative Computed Tomography (i-CT) integrated with 5-ALA fluorescence, and neuromonitoring-guided resection proved to safely increase the extent of resection [21,34]. We assume that, while the EIF algorithm might unveil residual tumour not clearly recognisable on the iCT, its confirmation should be achieved with the available intraoperative tools, especially in those cases in which a major influence of brain shift is suspected.

Regardless of tumour volume and location, the drawing of the resection cavity, as well as iCT image quality, appeared to be key. In terms of overall image quality, indeed, the worst results were observed when key anatomical structures (i.e., ventricles) were hardly distinguishable and the surgical cavity collapsed due to several factors (re-expansion of the surrounding parenchyma, gravity, etc.). In these cases, indeed, the manual drawing of the resection cavity might be challenging, and inaccuracies in tumour boundary delineation result in a “false positive” residual tumour. Autonomous drawing based on generative artificial intelligence, or a standardised step-by-step drawing protocol will be needed to address this problem and improve the software’s reliability.

Finally, regarding the analysis of navigation reliability, the registration of accuracy performed via the landmark-based measurements of the Target Registration Error after RIF and EIF revealed no significant change in control structures. Conversely, a statistically significant reduction towards lower TRE values was observed for lateral and cranio-caudal shift, Evan’s index, and midline shift. Our results are in line with those presented by Riva et al. [8] and Mazzucchi et al. [10], who reported reduced TRE values after EIF, with no significant change in TRE measurements for control structures as well. Overall, the elastic fusion algorithm proved to increase navigation accuracy by reducing the deformation occurring in both the parenchyma and ventricular system compared to standard rigid fusion algorithms [35].

### Limitations and Future Directions

This work has some limitations. First, the limited amount of data might have weakened the strength of the statistical analysis. Larger studies with the integration of multi-centre data are needed to provide surgeons with an effective and validated tool to manage a highly complex phenomenon like brain shift. A great bias is the consequence of measuring fusion accuracy by determining the TRE, which is dependent on iCT interpretation.

Similarly, as drawing the resection cavity to perform Virtual iMRI is operator-dependent, the software still lacks a standardised step-by-step drawing protocol, which may limit its reproducibility. Autonomous drawing based on generative artificial intelligence could, in the future, address this problem. Additionally, the availability of new CT devices with a software that reduces metal artifacts and noise might increase the iCT image quality, leading to the easier drawing of surgical cavity and elastic fusion itself. Nonetheless, such a leap forward is still long to come because, at present, applications in image interpretation are lagging behind compared to text analysis.

## 5. Conclusions

This study reports our early experience in the clinical feasibility, safety, and reliability of a novel EIF algorithm (Virtual iMRI, Brainlab) and proposes a new approach for the intraoperative evaluation of residual tumour in glioblastoma surgery. It also sheds light on factors which influence the “virtual” image. The algorithm proved to globally enhance the spatial image fusion, increasing neuronavigation accuracy.

## Figures and Tables

**Table 1:** Inclusion and exclusion criteria.

Inclusion Criteria	Exclusion Criteria
Diagnosis of glioblastoma	Different histological diagnosis
Supratentorial localisation	Infratentorial localisation
Neuroradiological dataset including pre- and post-operative MRI, intraoperative CT	Unavailable imaging dataset
Gross/Subtotal surgical excision	Surgical procedure different from gross/subtotal resection: biopsy, external ventricular drainage (DVE), ventriculoperitoneal shunt (DVP)

**Table 2:** Summarised quantitative variables expressed as mean and standard deviation (SD) or median and interquartile range (IQR) based on their distribution. Legend: NTR: necrosis/tumour ratio; EOTR: extent of resection.

VARIABLE	Frequency, Mean (SD), or Median (IQR) *n* = 80 Patients
Male	44
Female	36
Mean age (years)	61 years
Median craniotomy size (mm)	70 (21.5)
Head positioning	
0°	9
30°	16
45°	17
90°	21
Prone	12
Tumour location	
Frontal lobe	22
Parietal lobe	10
Temporal lobe	18
Occipital lobe	3
Insula	12
Central core	4
Median tumour volume on pre-operative Gd-enhanced MRI (cm^3^)	33.5 (33.9)
Median necrosis volume (cm^3^)	10 (20.4)
Median NTR (with 0 meaning that tumour was entirely fleshy and 1 fully necrotic or colloid)	0.37 (0.44)
Median tumour residual volume on Virtual iMRI (cm^3^)	1.88 (7.7)
Median tumour volume on post-operative Gd-enhanced MRI (cm^3^)	0 (0.8)
Mean EOTR	97%

**Table 3:** Confusion matrix reporting the FP: false positive; FN: false negative; TP: true positive; TN: true negative; PPV: positive predictive value; NPV: negative predictive value of iCT and Virtual iMRI. These data were utilised to calculate the diagnostic sensitivity and specificity of each imaging tool.

	TC	Virtual iMRI
FP	0	27
FN	13	0
TP	17	26
TN	50	27
SENSITIVITY	0.56	1
SPECIFICITY	1	0.5
PPV	1	0.49
NPV	0.79	1

**Table 4:** Target Registration Error (TRE) values reported between pre-operative MRI and iCT (after RIF) and between Virtual iMRI and iCT (after EIF) of paired anatomical landmarks. Note that Evan’s index refers to bifrontal ventricular diameter.

	Maximum		Minimum		Mean ± SD	
	RIF	EIF	RIF	EIF	RIF	EIF
**Lateral cortical shift**	32	22	0	0	12.5 ± 8.9	9.6 ± 6.8
**Cranio-caudal cortical shift**	30	22	0	0	10.9 ± 7.7	7.8 ± 5.6
**Anterior communicating artery**	3	5	−5	−4	−0.8 ± 1.6	−0.1 ± 1.4
**Basilar apex**	2	2	−2	−3	−0.3 ± 0.8	−0.1 ± 0.8
**D Midline shift**	9	7	−4	−6	1.5 ± 3	1 ± 2.6
**D Evan’s index**	19	11	−3	−10	4.8 ± 4.5	2.1 ± 3.8

## Data Availability

The data presented in this study are available upon request from the corresponding author due to privacy concerns.
